# The Evidence for Perioperative Anesthetic Techniques in the Prevention of New-Onset or Recurrent Complex Regional Pain Syndrome in Hand Surgery

**DOI:** 10.3390/jpm14080825

**Published:** 2024-08-04

**Authors:** Marcel Chua, Avinassh Ratnagandhi, Ishith Seth, Bryan Lim, Jevan Cevik, Warren M. Rozen

**Affiliations:** 1 Department of Surgery, Central Clinical School, Faculty of Medicine, Nursing and Health Sciences, Monash University, The Alfred Centre, 99 Commercial Road, Melbourne, VIC 3004, Australia; jratnagandhi@phcn.vic.gov.au (A.R.); ishith.seth@monash.edu (I.S.); bryan.lim@wh.org.au (B.L.); jevan.cevik@monash.edu (J.C.); warren.rozen@monash.edu (W.M.R.); 2 Department of Plastic and Reconstructive Surgery, Peninsula Health, 2 Hastings Road, Frankston, VIC 3199, Australia; 3 Monash Doctors Workforce, Monash Health, 246 Clayton Road, Clayton, VIC 3168, Australia; 4 Department of Plastic and Reconstructive Surgery, Western Health, 160 Gordon Street, Footscray, VIC 3011, Australia

**Keywords:** pain, plastic surgery, nerve, trauma, hand, patient outcomes

## Abstract

Complex regional pain syndrome (CRPS) is a multifaceted condition characterized by chronic neuropathic pain, allodynia, and hyperalgesia. The incidence of CRPS postoperatively is alarmingly high, particularly following carpal tunnel surgeries, Dupuytren’s fasciectomy, and repairs of wrist and hand fractures, with recurrence rates soaring in individuals with a history of CRPS. Despite extensive research, the management of CRPS remains complicated, highlighting the urgent need for effective prevention strategies. This scoping review aimed to consolidate current evidence surrounding the efficacy of perioperative anesthetic techniques in preventing new-onset or recurrent CRPS, focusing on the application of various anesthetic interventions. Through a comprehensive literature search, eight articles were identified, discussing a spectrum of techniques, including wide awake local anesthesia no tourniquet (WALANT) and various regional blockade methods. This review revealed that the WALANT technique, with its simplicity and lower costs, exhibited promising results in preventing CRPS. Conversely, techniques involving intravenous regional and axillary plexus blocks showed variable efficacy, necessitating further investigation. The scarcity of high-quality evidence underscores the critical need for meticulously designed, large-scale randomized controlled trials to validate these findings and explore the potential of stellate ganglion block in the prevention of recurrent CRPS.

## 1. Introduction

Complex regional pain syndrome (CRPS) is a cluster of sensory, autonomic, and motor disturbances to a particular body area. It can be divided into two types: CRPS Type I, where the syndrome occurs without a known nerve injury, and CRPS Type II, where the syndrome occurs in the presence of a specific nerve injury [1,2]. The salient feature of CRPS is chronic, persistent neuropathic pain, associated with allodynia and hyperalgesia [1,2]. Other features include swelling, stiffness, skin temperature dysregulation, skin color and texture changes, and abnormal sudomotor activity [1,2]. Diagnosis is guided by the Budapest CRPS diagnostic criteria, which are described in Table 1. In mild cases, symptoms may resolve by themselves; however, severe cases often suffer debilitating symptoms that can persist for years [1]. This can ultimately lead to loss of function and limit daily activities, dramatically affecting quality of life [3].

In the realm of hand surgery, there is a wide variation in the reported incidence of new-onset CRPS post-procedurally in at least 1.9% of carpal tunnel surgeries, at least 2% of Dupuytren’s fasciectomy, and at least 22% of repairs of wrist and hand fractures (e.g., distal radius, scaphoid, phalangeal, etc.) [1,5,6,7]. In patients with a previous episode of CRPS, recurrences after another hand procedure are likely to occur with their original severity, with incidence rates up to 73% in those with abnormal sympathetic function [8,9]. Despite a multitude of studies into the management of CRPS having been published, evidence is mostly poor to moderate quality, and its management remained complex [2,3]. Thus, prevention strategies became imperative but remained relatively unexplored apart from Vitamin C, to which large-scale reviews presented conflicting evidence [3,10].

The current understanding of the pathophysiology of CRPS was thought to involve the inflammatory process, nociception, and its association with sympathetic nervous system activity [2,5]. These processes can be targeted by anesthetic interventions such as neuraxial anesthesia, intravenous regional blockade (IVRB), local anesthetic sympathetic blockade (LASB), and brachial plexus blockade, as reported with low to moderate evidence by a Cochrane review for the management of CRPS [2]. As such, perioperative anesthesia was proposed as a potential option for preventing new-onset or recurrent CRPS. A recent systematic review has addressed this topic; however, it was not extensively reported as CRPS was measured secondarily to a broader topic of chronic pain [11]. This review aims to broaden the scope by exploring current evidence for perioperative anesthetic techniques in hand surgery, with a secondary focus on their role in preventing new-onset or recurrent CRPS. By consolidating available evidence, this review seeks to shed light on the potential for further large-scale studies to validate these findings.

## 2. Materials and Methods

### 2.1. Scoping Review

In consolidating all current evidence in a broad developing field, a scoping review was most appropriate, as its systematic approach to literature search attempted to maximize search results.

### 2.2. Literature Search and Selection

A comprehensive literature search was performed in multiple databases, including PubMed, Scopus, Google Scholar, and the Cochrane Central Register of Controlled Trials. To yield maximal search results for a topic with impoverished literature, the search extended from the database’s inception to December 2023. Keywords, medical subject headings (MeSH), and examples of search phrases used are listed in Supplementary Table S1. In Google Scholar, only the top 100 results were screened. Reference lists of initially included articles were cross-referenced to include additional articles not previously captured from databases.

Articles were included if they (A) were peer-reviewed articles available in the English language, (B) performed in the human population with no age limits, (C) described a perioperative anesthetic technique in upper extremity surgery, and (D) reported the incidence of CRPS [4]. Retracted articles were excluded from this review.

The entire literature search and article selection process was performed independently by the first two authors (Author 1 and Author 2). In the instance where the relevance of an article was unclear, a third author (Author 3) was consulted, and a consensus decision was reached.

### 2.3. Data Management and Extraction

Two independent authors (Author 1 and Author 2) extracted data into a standardized data extraction form. Discrepancies in data extraction were resolved by consensus and validated by a third author (Author 3). Data extracted included the study type, population (number and gender), procedure performed, anesthetic intervention modality and agent(s), tourniquet time, and the reported incidence of CRPS.

### 2.4. Assessment of Studies and Their Quality of Evidence

Due to the scarcity of articles, a narrative approach was taken to describe the incidence of CRPS with various anesthetic techniques. Randomized controlled trials (RCTs) were assessed for their risk of bias using the Risk of Bias (RoB) 2 tool; cohort studies were assessed using the Risk of Bias in Non-randomised Studies—of Interventions (ROBINS-1) tool; and case series were assessed using the Joanne Briggs Institute (JBI) critical appraisal tool [12,13,14].

## 3. Results

### 3.1. Included Articles

**Figure 1:**
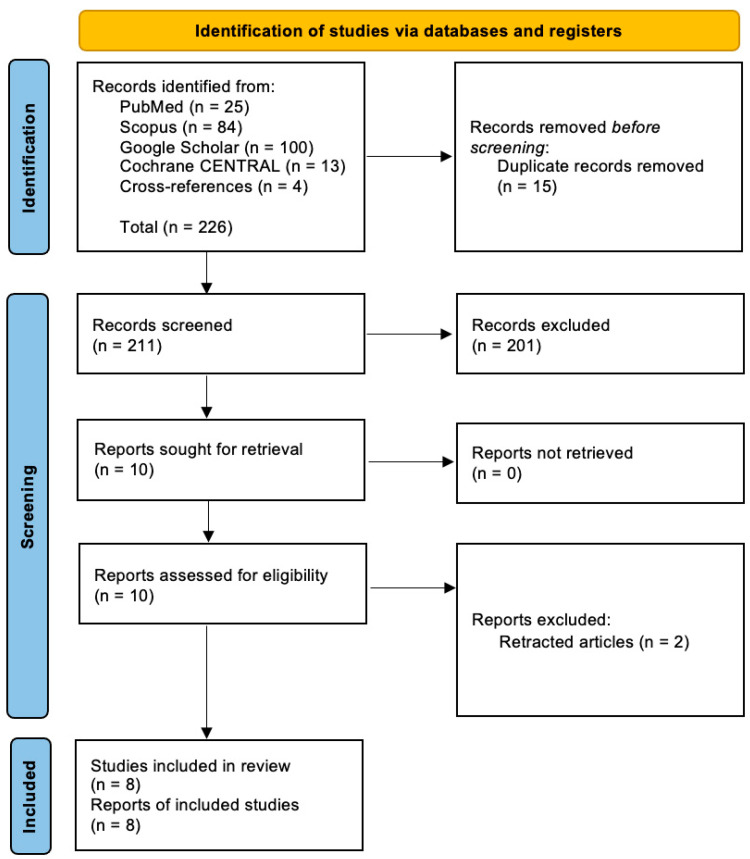
PRISMA flow diagram of literature search and screening of studies.

As illustrated in Figure 1, the literature search and selection process finally yielded eight articles. They consisted of three RCTs, two cohort studies, and three case series. Seven of these articles discussed new-onset CRPS, while the remaining one discussed recurrent CRPS.

### 3.2. Identified Techniques

In this review, a technique was defined as the mode of anesthesia administration (e.g., local anesthesia, regional blockade, axillary plexus block, etc.) along with its associated interventions (e.g., sedation) and/or pharmacologic agents (e.g., dexamethasone, clonidine, guanethidine, etc.). Although techniques could be broadly classified into the mode of anesthesia administration only, additional interventions and/or pharmacological agents were thought to confound results and therefore included as part of a unique technique.

Among the articles discussing new-onset CRPS, eight techniques were identified, including wide awake local anesthesia no torniquet (WALANT), WALANT with dexamethasone, local anesthesia with sedation, local anesthesia only, intravenous regional blockade (IVRB) with lidocaine alone, IVRB with lidocaine and clonidine, IVRB with guanethidine and axillary plexus block, and axillary plexus block alone [5,6,15,16,17,18,19]. In the article discussing recurrent CRPS, techniques identified include IVRB with lidocaine with post-surgical stellate ganglion block (SGB) with bupivacaine and IVRB with lidocaine only [20].

### 3.3. Incidence of CRPS Based on Various Anesthetic Techniques

The study characteristics, technique, and incidence of CRPS are summarized in Table 2. In new-onset CRPS, eight techniques were identified from seven studies. The WALANT techniques, with or without dexamethasone, appeared to be recently reported with zero cases of CRPS in three separate studies [15,17,18]. This incidence was consistent with another study by Far-Riera et al., who also reported an incidence of zero in their prospective analyses; however, in their retrospective analyses, they reported two cases (0.3%) of CRPS [16]. In a single study investigating the IVRB technique, anesthetic agent(s) with lidocaine alone or with lidocaine and clonidine were found to have no difference in affecting the incidence of CRPS (4.3%) [5]. A combined technique of IVRB with guanethidine and axillary plexus block resulted in the highest new-onset CRPS incidence among all studies (12.8%) [6]. The axillary plexus blockade was reported with a variation in CRPS incidence, ranging from zero to 11.3% among three studies [5,6,16].

In recurrent CRPS, two techniques were identified from a single study. A combined technique of IVRB and SGB resulted in a significant reduction in the incidence of recurrent CRPS (10.0%) compared to IVRB alone (72%).

### 3.4. Risk of Bias Assessment

Results of the risk of bias assessment of RCTs are depicted in Supplementary Table S2, cohort studies in Supplementary Table S3, and case series in Supplementary Table S4. All RCTs were assessed to have some to high overall risk of bias, similar to all cohort studies with moderate to serious overall risk of bias, according to their respective risk of bias assessment tools used. While the JBI critical appraisal used to assess case series does not summarize the overall risk of bias, we found at least some concerns in all case series.

The risk of bias arising in the measurement of the outcome was found in most RCTs and all cohort studies. Specifically, outcome assessors were not blinded to the interventions received [6,15,16,20]. This potentially results in bias favoring their interventions of interest (e.g., WALANT and IVRB) compared to controls. In the assessment of cohort studies, both studies were found to have serious bias due to confounding [16,20]. Both cohort studies reported potential confounders, including differences in gender proportion, age, surgery received, and tourniquet time; however, appropriate multivariate analyses have not been performed [16,20]. Similar concerns were also raised with case series in which confounders were not accounted for [17,18,19]. Furthermore, case series were unclear if appropriate criteria were used to diagnose CRPS, and thus presumed in this review.

## 4. Discussion

In this review, several techniques have been identified, and at first glance, the WALANT technique appeared to be the most promising in minimizing the incidence of CRPS; however, the critical appraisal results of these studies were found to have at least a moderate risk of bias [15,16,17,18]. Thus, the effectiveness of the WALANT technique cannot be validated currently. Likewise, for the IVRB and axillary plexus block techniques, there were at least some concerns with the evidence quality presented, and their relatively inferior results alone cannot render them obsolete. In summary, this review further reinforces the paucity of evidence in the prevention of CRPS and highlights the need for further studies.

In the prevention of new-onset CRPS, the WALANT technique, first implemented to reduce surgery waiting times, yielded a CRPS incidence rate of zero in three separate studies, with another retrospective study reporting two cases (0.3%) of CRPS [15,16,17,18]. Studies that employed alternate techniques (local anesthesia with sedation and axillary plexus block) also reported a CRPS incidence of zero and demonstrated no discernible statistical differences in other key markers, including the development of adverse effects, intraoperative pain, and postoperative pain. That said, the WALANT technique appeared to be advantageous over other techniques in terms of patient convenience and costs. This was evidenced by Far-Riera et al., who reported elevated levels of patient satisfaction in the WALANT group, attributing this to the absence of fasting requirements before the procedure and a shortened hospital stay [16]. Additionally, Far-Riera et al. reported average cost savings of USD 1025.34 in comparison to the axillary plexus block for carpal tunnel release and trigger finger surgery, possibly from the absence of an anesthetist review and investigations associated with regional anesthesia [16]. Similar trends were reported by Jerome 2023a, finding lower costs associated with WALANT when compared to the use of general anesthesia [18]. Finally, Far-Riera et al. highlighted a heightened patient turnover rate with the WALANT technique, enabling the completion of eight surgeries per day compared to the maximum of six surgeries achievable with the axillary plexus block [16]. This enhanced efficiency holds economic feasibility for medical institutions. A disadvantage, however, was reported by Ramos et al., who observed the occurrences of hematoma associated with the omission of a tourniquet in the WALANT technique [15]. Nevertheless, there was no statistical significance in the overall adverse effects between the WALANT and other techniques [15,16,17,18].

The IVRB technique was found to employ various combinations of anesthetic agents, including lidocaine alone, lidocaine with clonidine, or guanethidine alone [5,6]. Clonidine was initially thought to mitigate norepinephrine production, thereby attenuating sympathetic-induced pain mechanisms and reducing the incidence of CRPS [5]. However, the RCT by da Costa et al. found no differences in the incidence of CRPS with the addition of clonidine to lidocaine [5]. Guanethidine, initially explored by Gschwind et al. as a prophylactic agent for CRPS due to its ability to inhibit the binding of norepinephrine, yielded a relatively high CRPS incidence rate of 12.8% despite being accompanied by a shorter average tourniquet duration compared to those who did not develop CRPS [6]. Furthermore, guanethidine was associated with a spectrum of side effects, including seizures, hypotension, and nausea, often attributed to high doses and suboptimal tourniquet techniques [6]. Therefore, an IVRB technique with guanethidine would pose a precarious choice. That said, this finding could be biased as it is reported only by a single study, which included Dupuytren’s fasciectomies, a procedure relatively more invasive than carpal tunnel releases, trigger releases, ganglion cyst excision, etc.

The axillary plexus block was found to be an old technique described for upper limb surgery and continues to be used as a comparator to relatively newer techniques (e.g., WALANT and IVRB) [5,6,16]. Interestingly, the axillary plexus block appeared equally efficacious in preventing CRPS with an incidence of zero, similar to the WALANT technique in Far-Riera et al. [16]. In da Costa et al., despite a CRPS incidence of 11.3% in the axillary plexus block technique compared to 4.1% in the IVRB techniques being observed, no statistical differences were found [5]. In Gschwind et al., while considerably an outdated study, reported a lower CRPS incidence of 6.3% compared to the 11.3% in the combination of IVRB with guanethidine and axillary plexus block [6].

In the prevention of recurrent CRPS following hand surgery, only a single cohort study by Reuben, Rosenthal, and Steinberg evaluated the utilization of IVRB, both alone and in combination with stellate ganglion block (SGB) [20]. The IVRB in combination with SGB demonstrated a CRPS recurrence rate of 10%, markedly lower than the IVRB group recurrence rate of 72% [20]. The efficacy of the SGB technique performed immediately post-surgically was attributed to its inhibition of afferent nociceptive signals within the central nervous system, effectively diminishing the sympathetic drive of CRPS [20]. Furthermore, Reuben, Rosenthal, and Steinberg deliberated on an ideal timeframe for surgery following the resolution of first-onset CRPS. This study enrolled participants for surgery seven to eight months post-resolution of CRPS, resulting in a recurrence rate of 41% across all participants compared to 47% in a previous study of the knee enrolling participants five months post-resolution of CRPS [20,21]. That said, the ideal timeframe for surgery following the resolution of first-onset CRPS requires much further exploration, as the comparison of hand versus knee surgery above was inappropriate.

The quality of the evidence presented in this review is extremely limited because of its biasedness. As evaluated in the risk of bias assessment, studies that may be able to provide good evidence (e.g., RCTs and cohort studies) were assessed with moderate to high risk of bias. The statistical power, with a low number of cases presented in each article, further obscures the effectiveness of each technique. Compounding these biases further, most studies did not focus on CRPS as a primary outcome but rather reported it as a secondary outcome, leading to indirect evidence. Studying the association of an anesthetic technique to the risk of CRPS is also complex, given that the risk of CRPS is multifactorial, including patient demographics, co-morbidities, indication, complexity, and duration of surgery [1]. Additionally, postoperative factors, including quality of pain management, amount of active range of motion, and swelling control, also contribute to the risk of developing CRPS. These confounders were not accounted for in any of the included articles with appropriate analyses (e.g., multivariate multiple regression). Lastly, the findings of this review are not universally applicable to all types of hand surgeries. Most of the included studies focused on simple soft tissue surgeries like carpal tunnel release and Dupuytren’s contracture fasciectomy. As a result, the effectiveness of the anesthetic techniques discussed may not be applicable to more complex procedures such as arthrodesis, open fracture repairs, or tendon repairs of the hand. These limitations highlight that CRPS remains a significant challenge in pain medicine, and the current evidence on its management and prevention strategies in hand surgery is inadequate.

## 5. Conclusions

With the current poverty of strong evidence, the clinical application of anesthetic techniques in the prevention of new-onset or recurrent CRPS remains unready, therefore warranting further high-quality studies. The WALANT technique, demonstrating effectiveness in reducing incidences of CRPS post-surgically, lower cost, and quicker patient turnover compared to their alternatives, holds potential for further exploration. Alternative techniques, including the IVRB and axillary plexus block, also remain relevant for further studies, as current evidence is insufficient to render them obsolete. Future studies may be performed in the form of large-scale, meticulously planned RCTs that aim to address confounders to validate the efficacy of these techniques. In the prevention of recurrent CRPS, the poverty of literature calls for attention for researchers to explore this field. The stellate ganglion block currently holds value, and further review of this topic may help expand the breadth of this technique for future RCTs.

## Figures and Tables

**Table 1:** Budapest CRPS diagnostic criteria [4].

All the following statements must be met: - Continuing pain that is disproportionate to any inciting event. - At least one sign in ≥2 of the following categories. - At least one symptom in ≥3 of the following categories. - No other diagnosis can better explain the signs and symptoms.		
No.	Category	Signs/Symptoms
1	Sensory	Hyperalgesia (to pinprick) and/or allodynia (pain to light touch, deep somatic pressure, or joint movement).
2	Vasomotor	Temperature asymmetry, skin changes, and/or skin color asymmetry.
3	Sudomotor/edema	Edema, sweating changes, and/or sweating asymmetry.
4	Motor/trophic	Decreased range of motion, motor dysfunction (weakness, tremor, dystonia), and/or trophic changes (hair, skin, and nails).

**Table 2:** Study characteristics, techniques, and their incidence of CRPS [5,6,15,16,17,18,19,20].

Anesthetic Technique	Author(s)	No. of Patients	Follow-Up Period	Procedure(s)	Percentage of Females (%)	Mean Torniquet Time (min)	Phase of Administration	Anesthetic Agent	Incidence of CRPS (%)
New-Onset CRPS									
WALANT	Ramos et al., 2023 [15]	28	1 month	Carpal tunnel release/De Quervain’s tenosynovitis/synovial cyst/finger cyst/trigger finger	75.0	NA	Pre-incisional	9 mL of 1% lidocaine with 1:100,000 adrenaline, made up to 10 mL with 8.4% sodium bicarbonate	Zero
	Far-Riera et al., 2023 [16]	150 (prospective study)	1 month	Carpal tunnel release/trigger finger	69.0	NA		1% lidocaine with 1:100,000 adrenaline	Zero
		580 (retrospective study)	2–21 months (mean: 12 months)	Carpal tunnel release/trigger finger	65.0	NA			0.3% (two cases)
	Jerome, 2023a [17]	7	12–19 months (mean: 16.5 months)	Reconstruction of flexor pollicis longus ruptures following volar plate fixation of distal radius fractures.	14.3	NA		10 mL of 1% lidocaine with 1:100,000 adrenaline	Zero
WALANT with dexamethasone	Jerome, 2023b [18]	27	10–14 months (mean: 12.5 months)	Carmitz opponensplasty for carpal tunnel syndrome	81.5	NA		10 mL of 1% lidocaine with 1:100,000 adrenaline and 8 mg of dexamethasone	Zero
Local anesthesia with sedation	Ramos et al., 2023 [15]	28	1 month	Carpal tunnel release/De Quervain’s tenosynovitis/synovial cyst/finger cyst/trigger finger	85.7	NR		10 mL of 1% lidocaine only	Zero
Local anesthesia with torniquet	Lichtman, Florio and Mack, 1979 [19]	100	6 months	Carpal tunnel release	NR	16.0 ^		10 mL of 1% lidocaine only	5.0
IVRB with lidocaine alone	Da Costa et al., 2011 [5]	90	6 months	Carpal tunnel release	96.0	44.0 ± 9.4		40 mL of 0.5% lidocaine only	4.1
IVRB with lidocaine and clonidine	Da Costa et al., 2011 [5]	67	6 months	Carpal tunnel release	96.0	44.9 ± 10.7		40 mL of 0.5% lidocaine with clonidine 1 µg/kg	4.1
IVRB with guanethidine and axillary plexus block	Gschwind et al., 1995 [6]	39	8 weeks	Fasciectomy for Dupuytren’s contracture	10.3	82.0 ^		20 mL of fluid containing 20 mg of guanethidine only	12.8
Axillary plexus block	Far-Riera et al., 2023 [16]	150	1 month	Carpal tunnel release/trigger finger	69.0	NR		NR	Zero
	Da Costa et al., 2011 [5]	71	6 months	Carpal tunnel release	96.0	16.0 ± 8.4		30 mL of 2% lidocaine with 1:200,000 adrenaline	11.3
	Gschwind et al., 1995 [6]	32	8 weeks	Fasciectomy for Dupuytren’s contracture	18.8	70.0 ^		Not reported	6.3
Recurrent CRPS									
IVRB with lidocaine + SGB with bupivacaine	Reuben, Rosenthal and Steinberg, 2000 [20]	50	3 months	Upper extremity procedure including carpal tunnel release, tenolysis, tendon release, capsulotomy, arthrodesis and neuroma excision. Performed in those with a history of CRPS.	74.0	45.0 ± 18.0	Pre-incisional	40 mL of 0.5% lidocaine (IVRB) and 10 mL of 0.25% bupivacaine (SGB)	10.0
IVRB with lidocaine		50			82.0	40.0 ± 16.0	Immediately postoperative	40 mL of 0.5% lidocaine only	72.0

Abbreviations: WALANT = wide awake local anesthesia no torniquet; IVRB = intravenous regional blockade; NA = not applicable; NR = not reported. ^ Standard deviation not reported.

## Data Availability

The original contributions presented in the study are included in the Supplementary Material, further inquiries can be directed to the cooresponding author.

## Supplementary Materials

The following supporting information can be downloaded at: https://www.mdpi.com/article/10.3390/jpm14080825/s1, Table S1: List of keywords and medical subject headings used in systematic search; Table S2: Risk of bias assessment of randomized controlled trials using the RoB2 tool [5,6,15]; Table S3: Risk of bias assessment of cohort studies using the ROBINS-I tool [16,20]; Table S4: Risk of bias assessment of case studies using the JBI critical appraisal tool [17,18,19].

## References

[1] Ratti C., Nordio A., Resmini G., Murena L. (2015). Post-traumatic complex regional pain syndrome: Clinical features and epidemiology. Clin. Cases Min. Bone Metab..

[2] Ferraro M.C., Cashin A.G., Wand B.M., Smart K.M., Berryman C., Marston L., Moseley G.L., McAuley J.H., O’Connell N.E. (2023). Interventions for treating pain and disability in adults with complex regional pain syndrome—An overview of systematic reviews. Cochrane Database Syst. Rev..

[3] Saed A., Neal-Smith G., Fernquest S., Bourget-Murray J., Wood A. (2023). Management of complex regional pain syndrome in trauma and orthopaedic surgery—A systematic review. Br. Med. Bull..

[4] Harden N.R., Bruehl S., Perez R., Birklein F., Marinus J., Maihofner C., Lubenow T., Buvanendran A., Mackey S., Graciosa J. (2010). Validation of proposed diagnostic criteria (the “Budapest Criteria”) for Complex Regional Pain Syndrome. Pain.

[5] da Costa V.V., de Oliveira S.B., Fernandes M.d.C.B., Saraiva R.Â. (2011). Incidence of Regional Pain Syndrome after Carpal Tunnel Release. Is there a Correlation with the Anesthetic Technique?. Braz. J. Anesthesiol..

[6] Gschwind C., Fricker R., Lacher G., Jung M. (1995). Does Peri-Operative Guanethidine Prevent Reflex Sympathetic Dystrophy?. J. Hand Surg..

[7] Goh E.L., Chidambaram S., Ma D. (2017). Complex regional pain syndrome: A recent update. Burn. Trauma.

[8] Ackerman W.E., Ahmad M. (2008). Recurrent Postoperative CRPS I in Patients With Abnormal Preoperative Sympathetic Function. J. Hand Surg..

[9] Rocco A.G. (2005). Comment on: Abnormal contralateral pain responses from an intradermal injection of phenylephrine in a subset of patients with complex regional pain syndrome (CRPS), Mailis-Gagnon and Bennet. Pain.

[10] Seth I., Bulloch G., Seth N., Siu A., Clayton S., Lower K., Roshan S., Nara N. (2022). Effect of Perioperative Vitamin C on the Incidence of Complex Regional Pain Syndrome: A Systematic Review and Meta-Analysis. J. Foot Ankle Surg..

[11] Droog W., Walbeehm E.T., Konijn J.B., Lucas B.M.J., Coert J.H., Stolker R.J., Galvin E.M. (2023). A Systematic Review on Long-Term Postsurgical Pain Outcomes; What Is the Effect of Upper Extremity Regional Anesthesia?. Anesth. Analg..

[12] Higgins J.P.T., Altman D.G., Gøtzsche P.C., Jüni P., Moher D., Oxman A.D., Savović J., Schulz K.F., Weeks L., Sterne J.A.C. (2011). The Cochrane Collaboration’s tool for assessing risk of bias in randomised trials. BMJ.

[13] Sterne J.A., Hernán M.A., Reeves B.C., Savović J., Berkman N.D., Viswanathan M., Henry D., Altman D.G., Ansari M.T., Boutron I. (2016). ROBINS-I: A tool for assessing risk of bias in non-randomised studies of interventions. BMJ.

[14] Munn Z., Barker T.H., Moola S., Tufanaru C., Stern C., McArthur A., Stephenson M., Aromataris E. (2020). Methodological quality of case series studies: An introduction to the JBI critical appraisal tool. JBI Evid. Synth..

[15] Ramos P.R., Sakata R.K., Ribeiro H.C., Bonfanti A., Ferraro L. (2023). A prospective, comparative study of the analgesic effect between the WALANT technique and local anesthesia associated with sedation for hand surgery. Acta Cir. Bras..

[16] Far-Riera A.M., Perez-Uribarri C., Serrano M.J.E., González J.M.R. (2023). Impact of WALANT Hand Surgery in a Secondary Care Hospital in Spain. Benefits to the Patient and the Health System. J. Hand Surg. Glob. Online.

[17] Jerome J.T.J. (2023). Wide-awake local anesthesia no tourniquet and dexamethasone (WALANT-D) for modified Camitz opponens plasty in severe carpal tunnel syndrome—A retrospective study of 30 cases. J. Clin. Orthop. Trauma.

[18] Jerome J.T.J. (2023). Wide-awake local anesthesia No tourniquet (WALANT) for reconstruction of flexor pollicis longus ruptures following volar plate fixation of distal radius fractures—A Case series. J. Orthop. Rep..

[19] Lichtman D.M., Florio R.L., Mack G.R. (1979). Carpal tunnel release under local anesthesia: Evaluation of the outpatient procedure. J. Hand Surg..

[20] Reuben S.S., Rosenthal E.A., Steinberg R.B. (2000). Surgery on the affected upper extremity of patients with a history of complex regional pain syndrome: A retrospective study of 100 patients. J. Hand Surg..

[21] Katz M.M., Hungerford D.S. (1987). Reflex sympathetic dystrophy affecting the knee. J. Bone Jt. Surg. Br. Vol..

