# Supplementary material for: The Evidence for Perioperative Anesthetic Techniques in the Prevention of New-Onset or Recurrent Complex Regional Pain Syndrome in Hand Surgery
Source: J Pers Med. 2024 Aug 4;14(8):825. doi: 10.3390/jpm14080825 (PMC11355343; doi:10.3390/jpm14080825)
Supplement: Supplementary file 1 [file jpm-14-00825-s001.zip › jpm-3086522-supplementary.pdf]

Journal of *Personalized Medicine*

MDPI

Review

The Evidence for Perioperative Anesthetic Techniques in the Prevention of New-Onset or Recurrent Complex Regional Pain Syndrome in Hand Surgery

Marcel Chua [1,2,3,*], Avinassh Ratnagandhi [1,2], Ishith Seth [1,2], Bryan Lim [1,2,4], Jevan Cevik [1,2] and Warren M. Rozen [1,2]

Supplemental

Table S1. List of keywords and medical subject headings used in systematic search.

Keywords	Medical Subject Headings
Complex regional pain syndrome	Complex regional pain syndromes
Perioperative anesthesia	Reflex sympathetic dystrophy
Preoperative anesthesia	Nerve block
Intraoperative anesthesia	Anesthesia
Anesthesia	Anesthesiology
Hand surgery	Anesthetics, local
Upper extremity	Preoperative care
Carpal tunnel release	Perioperative care
	Hand
	Hand surgery
	Orthopedic procedures
	Orthopedics
	Carpal tunnel syndrome
	Decompression, surgical
	Dupuytren contracture
**Databases**	**Search Phrases**
PubMed	(complex regional pain syndromes[MeSH Terms]) AND ((nerve block[MeSH Terms]) OR (anesthesia[MeSH Terms]) OR (anesthesiology[MeSH Terms]) OR (anesthetics, local[MeSH Terms]) OR (preoperative care[MeSH Terms]) OR (perioperative care[MeSH Terms])) AND ((hand surgery[MeSH Major Topic]) OR (orthopedic procedures[MeSH Terms]) OR (carpal tunnel syndrome[MeSH Terms]) (decompression, surgical[MeSH Terms]) OR (dupuytren contracture[MeSH Terms]))
Scopus	TITLE-ABS-KEY (complex AND regional AND pain AND syndrome) AND TITLE-ABS-KEY (anesthesia) AND TITLE-ABS-KEY (hand AND surgery)

Table S2. Risk of bias assessment of randomized controlled trials using the RoB2 tool [5,6,15].

Author(s)	Risk of Bias Arising from the Randomization Process	Risk of Bias Due to Deviations from the Intended Interventions	Missing Outcome Data	Risk of Bias in Measurement of the Outcome	Risk of Bias in Selection of the Reported Result	Overall Risk of Bias Judgement

Ramos et al., 2023 [15]	Low	Low	Low	High	High	High
Da Costa et al., 2011 [5]	High	Low	Low	Low	Low	Some concerns
Gschwind et al., 1995 [6]	Low	Low	Low	High	Low	Some concerns

Table S3. Risk of bias assessment of cohort studies using the ROBINS-I tool [16,20].

**Author(s)**	**Bias Due to Confounding**	**Bias in Selection of Participants into the Study**	**Bias in Classification of Interventions**	**Bias Due to Deviations from Intended Interventions**	**Bias Due to Missing Data**	**Bias in Measurement of Outcomes**	**Bias in Selection of the Reported Result**	**Overall Risk of Bias Judgement**
Far-Riera et al., 2023 [16]	Serious	Low	Low	Low	Low	Moderate	Low	Moderate
Reuben, Rosenthal, and Steinberg., 2000 [20]	Serious	Low	Moderate	Low	Low	Serious	Low	Serious

Table S4. Risk of bias assessment of case studies using the JBI critical appraisal tool [17–19].

	**Were There Clear Criteria for Inclusion in the Case Series?**	**Was the Condition Measured in a Standard, Reliable Way for All Participants Included in the Case Series?**	**Were Valid Methods Used for Identification of the Condition for All Participants Included in the Case Series?**	**Did the Case Series Have Consecutive Inclusion of Participants?**	**Did the Case Series Have Complete Inclusion of Participants?**	**Was There Clear Reporting of the Demographics of the Participants in the Study?**	**Was There Clear Reporting of Clinical Information of the Participants?**	**Were the Outcomes or Follow-Up Results of Cases Clearly Reported?**	**Was There Clear Reporting of the Presenting Sites'/Clinics' Demographic Information?**	**Was Statistical Analysis Appropriate?**
Jerome, 2023a [17]	Yes	Yes	Unclear	Yes	Yes	Yes	Yes	Yes	No	Yes
Jerome, 2023b [18]	Yes	Yes	Unclear	Yes	Yes	Yes	Yes	Yes	No	Yes
Lichtman, Florio, and Mack, 1979 [19]	Yes	Unclear	Unclear	Yes	No	No	No	Yes	No	Yes
